# Laser Ablated Nanocrystalline Diamond Membrane for Infrared Applications

**DOI:** 10.3390/s22030829

**Published:** 2022-01-22

**Authors:** Maxim S. Komlenok, Margarita A. Dezhkina, Vadim S. Sedov, Oleg A. Klimenko, Sergey A. Dyakov, Nikolay A. Gippius

**Affiliations:** 1 Prokhorov General Physics Institute of the Russian Academy of Sciences, Vavilova Street 38, 119991 Moscow, Russia; m.a.dezhkina@gmail.com (M.A.D.); sedovvadim@yandex.ru (V.S.S.); 2 Skolkovo Institute of Science and Technology, Bolshoy Boulevard 30, Bld. 1, 121205 Moscow, Russia; o.klimenko@skoltech.ru (O.A.K.); s.dyakov@skoltech.ru (S.A.D.); n.gippius@skoltech.ru (N.A.G.); 3 PN Lebedev Physical Institute of RAS, Leninskiy Prospekt 53, 119991 Moscow, Russia

**Keywords:** nanocrystalline diamond, photonic crystal slab, direct laser-writing, quasiguided modes, circular polarization

## Abstract

We are reporting on laser microstructuring of thin nanocrystalline diamond membranes, for the first time. To demonstrate the possibility of microstructuring, we fabricated a diamond membrane, of 9 μm thickness, with a two-dimensional periodic array of closely located chiral elements. We describe the fabrication technique and present the results of the measurements of the infrared transmission spectra of the fabricated membrane. We theoretically studied the reflection, transmission, and absorption spectra of a model structure that approximates the fabricated chiral metamembrane. We show that the metamembrane supports quasiguided modes, which appear in the optical spectra due to grating-assisted diffraction of the guided modes to the far field. Due to the C${}_{4}$ symmetry, the structure demonstrates circular dichroism in transmission. The developed technique can find applications in infrared photonics since diamond is transparent at wavelengths >6 μm and has record values of hardness. It paves the way for creation of new-generation infrared filters for circular polarization.

## 1. Introduction

Studying the possibility of controlling the polarization and spectral properties of metasurfaces in the infrared (IR) range is an important task for different applications, including the fabrication of multichannel IR emitters and narrow-band sources of thermal radiation, control of the polarization states of IR light (filtering) [1,2,3], chemical detection [4,5,6], radiation cooling [7,8,9], thermal photovoltaics [10,11,12,13,14], and thermal masking [15,16]. Simultaneous requirements for a high-quality factor of optical resonances of the metasurface and its mechanical strength significantly reduce the number of materials from which it can be made. For example, materials, such as KBr and NaCl, are transparent in the wavelength range up to ∼20 μm, but have very low strength; ZnSe and ZnS are transparent up to ∼15 microns, but their strength is still low; more traditional materials for photonics, such as Si and Ge, are quite strong, but they are transparent only up to ∼9 μm and ∼13 μm, respectively. Due to the transparency of diamond at wavelengths >6 μm [17] and record values of hardness, it is a promising material for IR photonics [18,19]. For IR applications mentioned above, we need to use the diamond membranes with an aperture of about 1 mm and with thickness providing the propagation of quasi-waveguide modes, which corresponds to the range of 5–9 μm. Specifically, nanocrystalline diamond (NCD) film is perfect for this goal.

As of now, there are only a few works in which the nanocrystalline diamond has been used to create devices designed to operate in the IR range. Moreover, none of these elements were based solely on an NCD film—all were hybrid devices. In references [20,21], the authors report on the development of composite diamond film for a short-mm wave range and thin-diamond windows for THz traveling wave tube. In them, Ming et al. created a structure consisting of alternating layers of an ultra-nanocrystalline diamond (UNCD) and polycrystalline diamond to reduce the roughness of existing polycrystalline windows and prevent air leakage. Remes et al. [22] used nanocrystalline diamond coatings for infrared planar waveguides to reduce optical losses, proposing a structure based on hydrogenated amorphous silicon (a-Si: H) coated by very thin NCD film. In [23], single-mode suspended ridge waveguides on a silicon-NCD platform operating in a wide range of wavelengths covering 2.5–16 μm were created. Nanocrystalline diamond-coated silicon elements for infrared attenuated total reflection spectroscopy (ATRS) were proposed by Arndt et al. [24]. This team used NCD coatings to widen the applications of ATR spectrometers into an industrial sphere, where their elements can be exposed to abrasive components and aggressive cleaning agents. Another group used nitrogen- and boron-doped NCD films as reflective surfaces in fiber-optic interferometric sensors designed for the measurement of the refractive index of liquids [25]. A team of scientists from the Okinawa Institute of Science and Technology Graduate University created a nanocrystalline diamond-glass platform for future biomedical and quantum technological applications [26].

To create an optical element entirely based on nanocrystalline diamond film, precise tools for its microprocessing are required. One of these tools is laser radiation, which is proven to be an effective instrument for creating elements of photonics and electronics based on diamond [27,28]. As a result of local laser action, graphitized material is formed on the surface or in the bulk of a diamond (depending on irradiation conditions). This method allows obtaining structures that have opposite optical and electrical properties of diamond and graphite and is already successfully used, for example, to design detectors of ionizing radiation [29,30,31,32] and photonic crystals [17] based on single crystal diamond, and optical elements of the terahertz range [33,34] based on polycrystalline diamond. However, despite the presence of works dedicated to the studies of laser-induced changes in conductivity [35], and optical properties [35,36], and of laser-induced ablation of nanocrystalline diamond films [37,38], laser radiation was not used to make optical elements based on NCD. Here, we propose to use laser-induced surface structuring of an NCD film for this goal. Unfortunately, due to the difference in density of the original and modified by laser radiation materials, a local transformation of diamond into graphite can lead to cracking [39,40]. It is crucial in our case, where we need to modify the surface by fabricating structures with a depth of several microns in such a thin membrane of the large aperture, as mentioned above, which is challenging.

In this work, we studied a diamond metamembrane with "chiral morphology" of surface patterns. It is well known that chiral structures have great possibilities in circular polarization filtering (see [41,42,43,44,45,46,47,48,49,50]). One example involves three-dimensional chiral photonic crystals that show giant circular dichroism in the near-infrared range due to their polarization-dependent photonic stop bands [47,48,49]. Although such structures have a relatively wide operation range, they require a complicated fabrication process. One can use a chiral metasurface to overcome this problem due to only a single lithography layer. The transmission properties of metasurfaces are determined by the type of symmetry [51]. Structures with C${}_{2}$ symmetry demonstrate strong circular dichroisms in transmission [52]. It has been shown that the chiral silicon metasurfaces [1], arrays of silver zigzag stripes [53], as well as more complex C${}_{2}$ symmetrical structures, such as a gold pixelized metasurface [2], can be used to obtain the circularly polarized light in the near- and middle-infrared. Unlike C${}_{2}$ symmetrical structures, metasurfaces with C${}_{4}$ symmetry demonstrate circular dichroism in transmission along the normal direction to the sample surface [52]. In references [41,42,43,44,45], the authors used C${}_{4}$ symmetrical metasurfaces to obtain the circularly polarized visible and near-infrared photoluminescence of semiconductor quantum dots. Due to the scalability of Maxwell’s equations, the wavelengths of optical resonances in C${}_{4}$ symmetrical metasurfaces can be shifted to the middle infrared by increasing their thickness and period.

In this work, we demonstrate an approach that enables us to create the first optical element for mid-IR radiation entirely made of a nanocrystalline diamond membrane. We also experimentally and theoretically study the optical properties of the fabricated membrane and demonstrate the appearance of photonic quasiguided modes in it.

## 2. Experimental

The synthesis of the NCD film was carried out on a monocrystalline silicon substrate with the (001) orientation and size of 10×10×0.35 mm${}^{3}$. To stimulate diamond nucleation, detonation diamond nanoparticles (average size 5 nm) from an aqueous suspension were applied to the substrate. The growth of the diamond phase was realized using chemical vapor deposition in microwave plasma in an ARDIS-100 reactor (2.45 GHz, 5 kW), in a hydrogen/methane/nitrogen gas mixture, at a total gas flow rate of 500 cm${}^{3}$/min (H${}_{2}$:460/CH${}_{4}$:20/N${}_{2}$:20), pressure in the chamber of 65 Torr, and microwave power of 4 kW. The substrate temperature was measured with a dual-beam pyrometer Micron M770. During the growth, the substrate temperature was maintained at a level of ∼900 ${}^{\circ }$C. The synthesis time was 3 h. The thickness of the grown NCD film was 9.0±0.5 μm.

**Figure 1:**
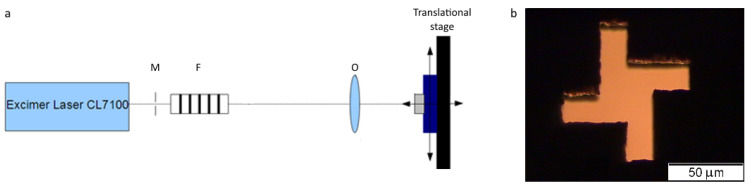
(**a**) Schematic of the projection optical setup used for sample irradiation; (**b**) the mask image obtained using an optical microscope.

Surface structuring was performed using a commercial excimer KrF (λ=248 nm) laser (CL7100, Optosystems Ltd.) with a pulse duration of τ=20 ns. The optical scheme of sample irradiation is shown in Figure 1a. Excimer laser radiation uniformly illuminated a pre-fabricated (by laser ablation) mask (Figure 1b) of the required shape (M), which was then projected onto the sample surface with a demagnification factor of 1:20 using an objective (O). The fluence was varied using a set of filters (F). The sample was fixed in a holder on a translational three-axis stage, making it possible to move it relative to the laser beam. Thus, the laser beam was scanned over the surface to form a periodic structure. The ablation rates of the NCD film were calculated at various laser fluences and the optimal conditions (20 J/cm${}^{2}$, 4 pulses) were determined for the structure fabrication on the film surface. A periodic structure with an area of 1.1×1.1 mm${}^{2}$ on the surface of the NCD film was created by direct laser writing. The depth of the ablated craters was 2500 nm. Laser ablation of the prepared membrane could cause the formation of cracks and splits for two reasons. The first reason is an increase of pressure in the irradiation spot due to the lower density of the graphitized material formed at the bottom of the crater than the diamond. Second, this is a shock wave formation during laser ablation, which also causes pressure increase. One should also keep in mind the large depth of the craters relative to the thickness of the membrane. The silicon substrate supports a thin diamond film on the rear side and protects it from splits. Therefore, the diamond film on the silicon substrate was first irradiated, and then silicon was etched in the structured region to create the membrane. The initial silicon substrate was partly etched in a mixture of concentrated hydrofluoric (99%) and nitric (99.9%) acids (1:1) to obtain membranes in the structured and clear areas for the measurements of optical transmittance. After the etching, samples were cleaned in deionized water, and then in isopropanol (99.9%). The morphology of the structure was analyzed using an AxioTech 25HD Carl Zeiss optical microscope, a Zygo NewView 5000 interference microscope, and an Integra Spectra NT-MDT scanning probe microscope (SPM). Transmission spectra were measured in the range of 2–20 μm using a FTIR spectrometer Bruker VERTEX 70v with a linear polarizer.

## 3. Results and Discussion

**Figure 2:**
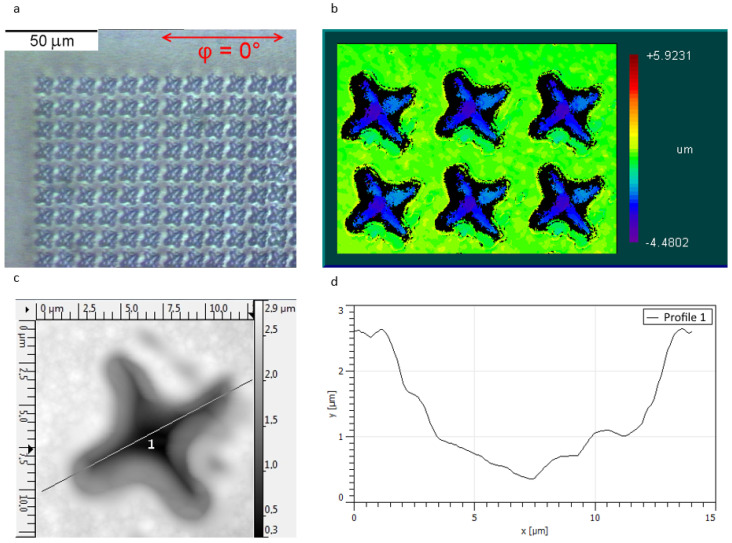
Image of a periodic structure, obtained with an optical microscope (**a**); interference microscope (**b**); scanning probe microscope (**c**); the profile of the chiral element measured by scanning the probe microscope (**d**).

In Figure 2a, the optical image of a fragment of the periodic structure is shown. No cracks or fractures of the membrane are observed. The 2D morphology map (Figure 2b) obtained by the interference microscope demonstrates the high reproducibility of the patterns on the NCD surface. The morphology of the single element was analyzed in detail by SPM (Figure 2c). The crater profile depicted in Figure 2d demonstrates the maximum depth of 2500 nm. However, the sides of the crater are not as vertical as desired.

**Figure 3:**
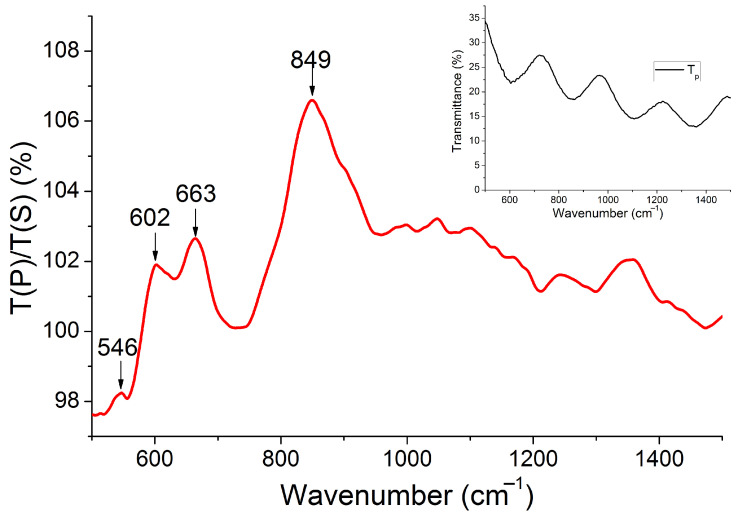
Ratio of the IR transmission spectra $T\left(\phi =90^{\circ }\right)/T\left(\phi =0^{\circ }\right)$. The spectrum at $\phi =0^{\circ }$ is shown in the inset.

To study the optical properties of the fabricated membrane experimentally, one can measure the transmission spectra since the diamond is transparent in the middle infrared range. In our experiment, we registered the transmission spectra at various polarization angles with respect to the sample structure orientation, ϕ (the polarization direction corresponding to $\phi =0{}^{\circ }$ is shown in Figure 2a). It enabled us to use the transmission spectrum measured at $\phi =0^{\circ }$ (inset in Figure 3) as a background for the transmission spectra measured at all other angles. The ratio of the transmission spectra measured at $\phi =90^{\circ }$ and $\phi =0^{\circ }$, $\eta_{\mathrm{linear}}=T\left(p\right)/T\left(s\right)$ (symbols p- and s- denote linear polarizations), is shown in Figure 3. It can be seen in Figure 3 that the measured function $\eta_{\mathrm{linear}}\left(\omega /c\right)$ within the entire measurement range of wavenumbers $\eta_{\mathrm{linear}}\left(\omega /c\right)$ is close to 1, but has peaks at 849, 663, 602, and 546 cm${}^{-1}$. At the same time, it is well known that C${}_{4}$ symmetrical structures should have identical transmission spectra in s- and p-polarizations at a normal angle of incidence and, hence, $\eta_{\mathrm{linear}}\left(\omega /c\right)$ should be constant and equal to 1. We attribute the effect of non-unity of $\eta_{\mathrm{linear}}$ to the small deviation of the incidence angle from normal.

**Figure 4:**
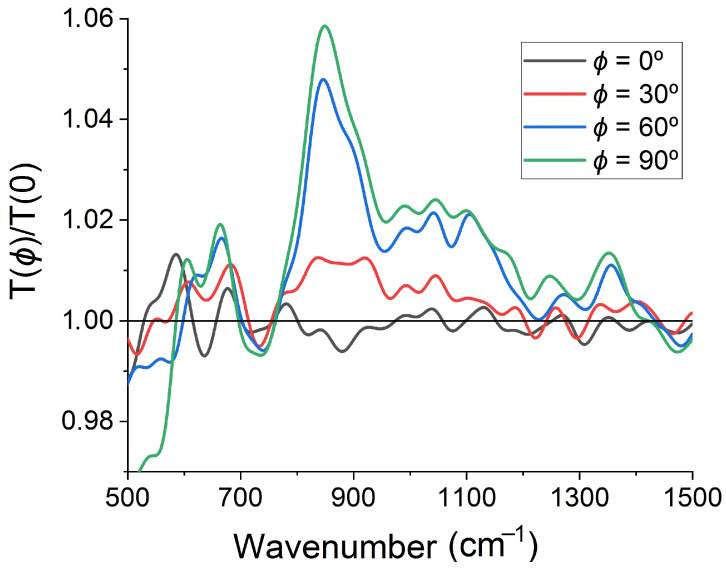
Ratio of the IR transmission spectra $T\left(\phi \right)/T\left(0^{\circ }\right)$ at polarization angles of $\phi =0^{\circ },\;30^{\circ },\;60^{\circ }$, and $90^{\circ }$.

**Figure 5:**
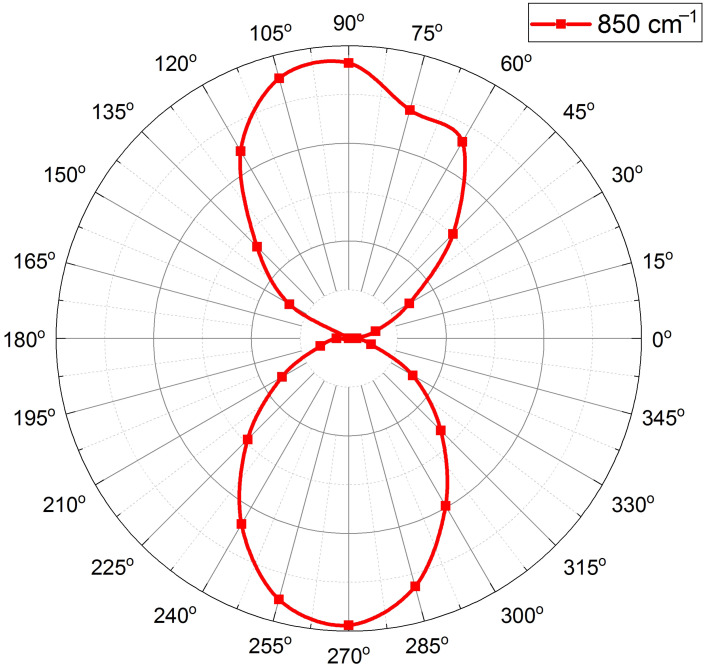
Effective polarization calculated for the given wavenumber 850 cm${}^{-1}$, as follows: $\left(T\left(\phi \right)/T\left(0^{\circ }\right)-min\left[T\left(\phi \right)/T\left(0^{\circ }\right)\right]\right)/\left(max\left[T\left(\phi \right)/T\left(0^{\circ }\right)\right]-min\left[T\left(\phi \right)/T\left(0^{\circ }\right)\right]\right)$.

The amplitude of the observed peaks in spectral ratio $T\left(\phi =90^{\circ }\right)/T\left(\phi =0^{\circ }\right)$ depends on the polarization angle, ϕ. One can see it in Figure 4, where we plot $T\left(\phi \right)/T\left(0^{\circ }\right)$ spectra obtained for various polarization angles in the numerator, $\phi =0^{\circ },\;30^{\circ },\;60^{\circ }$, and $90^{\circ }$, while keeping the same denominator, $T(\phi =0^{\circ })$. Angles $\phi =0^{\circ }$ and $180^{\circ }$ correspond to the constant spectral ratio $T\left(\phi \right)/T\left(0^{\circ }\right)=1$, whereas the maximum deviation from 1 is observed at angles $\phi =90^{\circ }$ and $270^{\circ }$. The angular dependence of the ratio $T\left(\phi \right)/T\left(0^{\circ }\right)$ has a well pronounced C${}_{2}$ symmetry caused by the non-normal incidence of the transmitted light. To show it, we fix the incident wavenumber at 850 cm${}^{-1}$, where the highest $T\left(\phi =90^{\circ }\right)/T\left(\phi =0^{\circ }\right)$ maximum is observed, and plot the normalized ratio $T\left(\phi \right)/T\left(0^{\circ }\right)$ as a function of ϕ (Figure 5). Since the deviation of $T\left(\phi \right)/T\left(0^{\circ }\right)$ from 1 is relatively small, we use the following normalization form $\left(T\left(\phi \right)/T\left(0^{\circ }\right)-min\left[T\left(\phi \right)/T\left(0^{\circ }\right)\right]\right)/\left(max\left[T\left(\phi \right)/T\left(0^{\circ }\right)\right]-min\left[T\left(\phi \right)/T\left(0^{\circ }\right)\right]\right)$ to reveal the angular features. The polarization axis direction of the normalized $T\left(\phi \right)/T\left(0^{\circ }\right)$ ratio corresponds to the angles $\phi =90^{\circ }$ and $270^{\circ }$.

**Figure 6:**
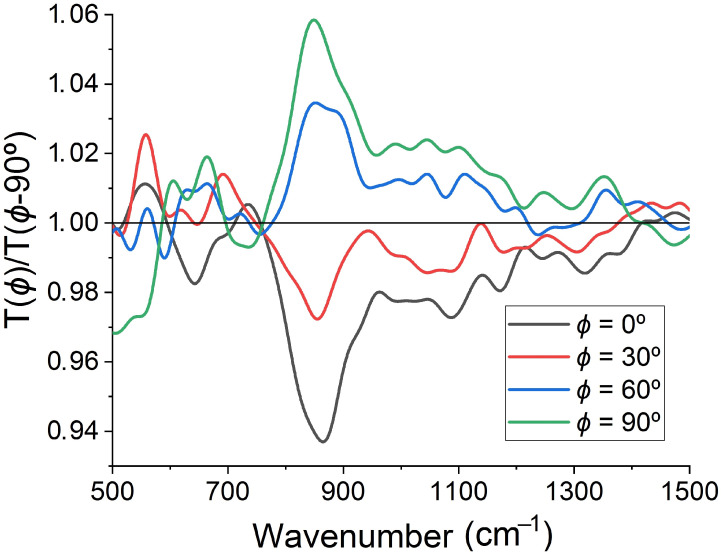
Ratio of the IR transmission spectra $T\left(\phi \right)/T\left(\phi -90^{\circ }\right)$ at polarization angles of $\phi =0^{\circ }$, $30^{\circ },\;60^{\circ }$, and $90^{\circ }$.

**Figure 7:**
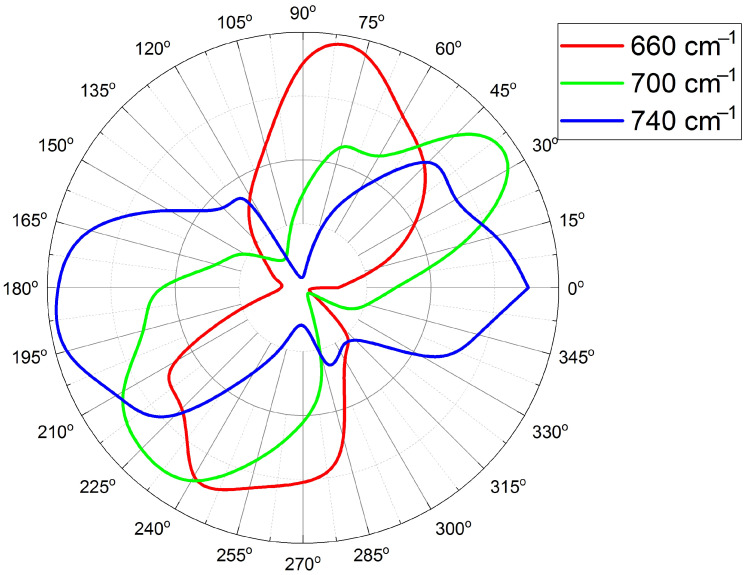
Effective polarization calculated for each given wavenumber (660, 700, and 740 cm${}^{-1}$), as: $\left(T\left(\phi \right)/T\left(\phi -90^{\circ }\right)-min\left[T\left(\phi \right)/T\left(\phi -90^{\circ }\right)\right]\right)/\left(max\left[T\left(\phi \right)/T\left(\phi -90^{\circ }\right)\right]-min\left[T\left(\phi \right)/T\left(\phi -90^{\circ }\right)\right]\right)$.

The angular dependence of the ratio of mutually perpendicular transmission spectra, $T\left(\phi \right)/T\left(\phi -90^{\circ }\right)$, also demonstrates the C${}_{2}$ symmetry, but it has some differences too. First, the constant spectral ratio $T\left(\phi \right)/T\left(0^{\circ }\right)=1$ appears at angles $\phi =45^{\circ },\;135^{\circ },\;225^{\circ }$ and $315^{\circ }$ and in a reduced spectral range (760–1400 cm${}^{-1}$). Second, the ratio $T\left(\phi \right)/T\left(\phi -90^{\circ }\right)$ not only exceeds 1 (having maxima at $\phi =90^{\circ }$ and $270^{\circ }$), but also goes below 1, achieving minima at $\phi =0^{\circ }$ and $180^{\circ }$ (see Figure 6). The most important effect is a continuous rotation of the polarization axis direction of the normalized $T\left(\phi \right)/T\left(\phi -90^{\circ }\right)$ ratio in the range 660–740 cm${}^{-1}$ (see Figure 7), which is a direct consequence of a circular dichroism of the studied nanocrystalline diamond membrane.

**Figure 8:**
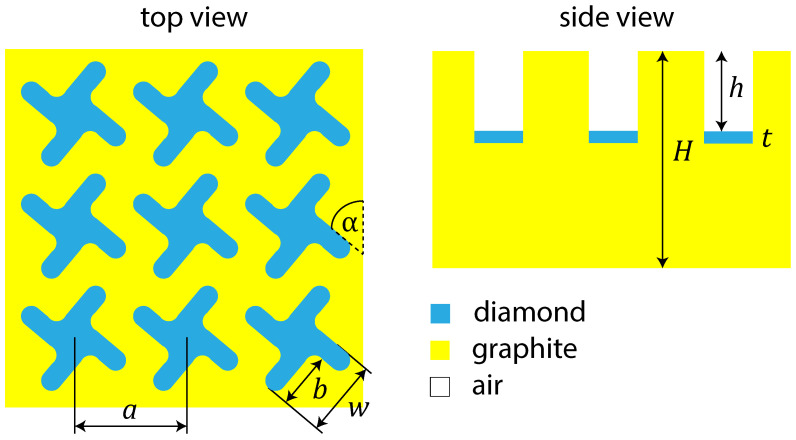
Schematic of a model structure. In the calculation, the following parameters are used: a=11.5μm, w=8.1μm, b=5.6μm, $\alpha =50^{\circ }$, H=9μm, h=2.5μm, t=100 nm.

To study the optical properties of the fabricated diamond metamembrane in more detail, and, particularly, to prove that the above effect of a non-unit $\eta_{\mathrm{linear}}$ is related to an inclined incidence of light, we are going to calculate the reflection, transmission, and absorption spectra. The corresponding model structure is shown in Figure 8. We assume that the model structure is perfectly periodic and has ideal smooth interfaces between materials. The graphite layer on the bottom of chiral elements has t=100 nm thickness and is bounded by parallel planes that are perpendicular to z-axis. In calculation, we also assume that the structure period is a=11.5 μm, the angle of the chiral element to y-axis is $\phi =50^{\circ }$, the entire membrane thickness is H=9 μm, and the etch depth is h=2.5 μm.

**Figure 9:**
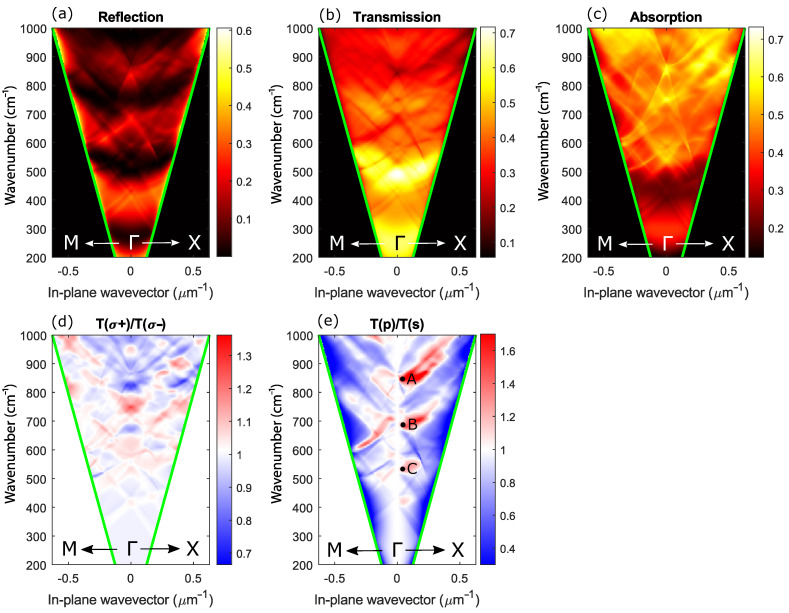
Wavenumber and in-plane wave vector dependence of (**a**) reflection coefficient, (**b**) transmission coefficient, (**c**) absorption coefficient, (**d**) ratio of transmission coefficients in $\sigma^{+}$ and $\sigma^{-}$ circular polarizations, (**e**) ratio of transmission coefficients in *p* and *s* linear polarizations. Green lines show the air light cones. The above coefficients are calculated along Γ-X and Γ-M photonic crystal directions. ω(A)/c≈851 cm${}^{-1}$, ω(B)/c≈690 cm${}^{-1}$, ω(C)/c≈540 cm${}^{-1}$.

To simulate the optical characteristics of the model structure, we used the Fourier modal method in the scattering matrix form [54]. This method is based on splitting a structure into elementary planar layers, homogeneous in the z-direction and 2D periodic in x- and y-directions. In this splitting, the cross-section of each chiral element is approximated by a staircase. The solutions of Maxwell’s equations for each layer are found by an expansion of the electric and magnetic fields into Floquet–Fourier modes (plane waves). The exact solution can be presented as an infinite series over these modes, in the limit of an infinite number of steps per chiral element. In a numerical simulation, the scattering matrices are determined by taking a finite number of stairs, $N_{s}$, and by truncating the Fourier series on a finite number of plane waves $N_{p}$. The calculation accuracy increases with $N_{s}$ and $N_{p}$; however, calculation time also increases. In order to improve the convergence, we implemented Li’s factorization rules [55]. As a result, we used 9 plane waves in both x- and y-directions, so that the total number of plane waves was $N_{g}=81$. The number of stairs per chiral element was $N_{s}=100$. Our calculation revealed that at these $N_{g}$ and $N_{s}$, the computation scheme numerically converges. Reflection, transmission, and absorption coefficients calculated by the Fourier modal method are shown in Figure 9 as a function of the in-plane wave vector and the wavenumber. In Figure 9, one can see that all spectra are characterized by the local peaks and dips, which can be separated into narrow and wide. The distance between the closest wide local maxima is ≈250 cm${}^{-1}$ that corresponds to the Fabry–Pérot oscillations on the entire 9 μm thickness of the membrane. These Fabry–Pérot oscillations are seen as interference fringes in the experimental transmission spectra shown in the inset of Figure 3. Against the background of the wide Fabry–Pérot maxima, the theoretical *R*, *T*, *A* spectra also have fine structures as high-dispersion narrow local maxima and minima. These are distinctive features of photonic crystal waveguides known as Wood’s anomalies. There are two types of Wood’s anomalies; both can be seen in Figure 9. The first type is Wood–Rayleigh anomalies, which appear at the wavenumber exactly corresponding to the opening of new diffraction channels. This type of anomaly is seen in the theoretical transmission spectrum (Figure 9b) as a bright spot at $k_{\|}=0$ and ω/c≈869.6 cm${}^{-1}$. This wavenumber corresponds to the condition for the first diffraction channel opening in air a=λ. Another type of Wood anomaly in the photonic crystal slabs is a Wood–Fano anomaly, which appears due to grating-assisted outcoupling of the guided modes to the far-field. Other commonly accepted names for these spectral features are quasiguided modes or quasi-normal guided modes [56,57,58]. Due to the diffraction on periodicity, the optical spectra measured in far-field have peaks corresponding to quasiguided modes because the dispersion curves of the original guided modes of a homogeneous waveguide fold back to the first Brillouin zone forming families of modes [59], one is seen as a dip in Figure 9b at $k_{\|}=0$ and ω/c≈550 cm${}^{-1}$. The wavenumber of this quasiguided mode is lower than that of the Wood–Rayleigh anomaly at $k_{\|}=0$ because the guided modes of the original homogeneous waveguide are below the vacuum light line. Wood–Fano anomalies have a Fano-like shape in the optical spectra. This is because the incoming field interacts with a background set by a modal structure of the diamond metamembrane [60,61].

Another important feature of our structure is its surface profile’s chirality and four-fold rotational symmetry. Due to its chirality, our metamembrane has circular dichroism in the reflection, and transmission spectra [62,63]. Figure 9d shows the theoretical ratio of the transmission coefficients calculated in different circular polarizations, $\eta_{\mathrm{circ}}=T\left(\sigma^{+}\right)/T\left(\sigma^{-}\right)$. It can be seen from Figure 9d that the maximal and minimal values of the parameter $\eta_{circ}$ are ≈1.35 and ≈0.65. One can further optimize the geometrical parameters of the metamembrane in such a way that it can work as a perfect filter, transmitting only one circular polarization at the desired wavenumber.

Regarding linear polarization, due to the C${}_{4}$ symmetry, our metamembrane should have identical transmission spectra in s- and p-polarizations at a strictly normal angle of incidence (see the vertical white region at $k_{\|}=0$ in Figure 9e). With an increase of the incidence angle, the four-fold symmetry is broken, and the structure transmits two linear polarizations differently. It explains a non-unity of the measured ratio $\eta_{\mathrm{linear}}=T\left(p\right)/T\left(s\right)$ of our C${}_{4}$ symmetrical metamembrane provided that in the experiment, the actual angle of incidence slightly deviates from normal. The calculated local maxima of $\eta_{\mathrm{linear}}$ have spectral positions at 851, 690, and 540 cm${}^{-1}$, which is quite close to the experiment.

**Figure 10:**
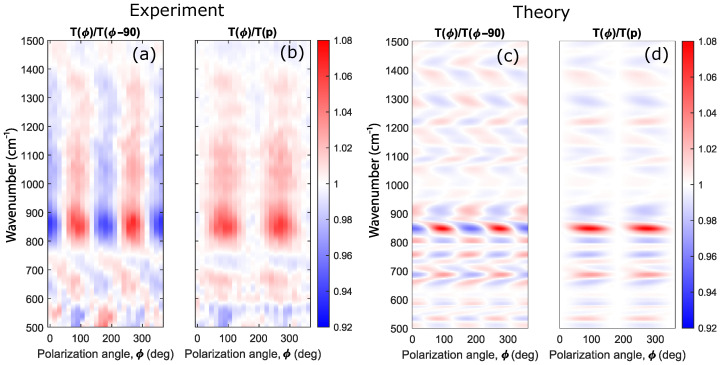
Experimental (**a**,**b**) and theoretical (**c**,**d**) dependencies of the $T\left(\phi \right)/T\left(\phi -90^{\circ }\right)$ (**a**,**c**) and T(ϕ)/T(p) (**b**,**d**) on the angle of polarization ϕ and the wavenumber ω/c. Calculations are made for the polar angle of incidence $\theta =1^{\circ }$. The color scale is shown on the right.

The experimentally measured ratios $T\left(\phi \right)/T\left(\phi -90^{\circ }\right)$ and T(ϕ)/T(p) as well as their theoretical counterparts are shown in Figure 10 as the functions of the polarization angle ϕ and the wavenumber ω/c. In Figure 10, both these functions have a 180${}^{\circ }$ period, which is dictated by the system’s symmetry, taking into account a non-normal incidence of light in measuring the transmission spectra. The maximal amplitude is achieved at the quasiguided mode wavenumber ≈850 cm${}^{-1}$ (point A in Figure 9e). Although the theoretical and experimental spectral peak positions and the periods agree with each other, the resonance widths in the experimental spectra are wider than those in the theoretical ones. It is a common situation for non-ideal photonic structures where scattering loss on imperfections and roughness plays a significant role.

## 4. Conclusions

The possibility of non-destructive laser microstructuring of thin diamond membranes was demonstrated. The optimal irradiation parameters for fabricating a structure of a given size have been determined. Following the calculations performed, a periodic chiral structure with four-fold rotation symmetry was created by the direct laser drawing on the surface of the membrane. The measured IR transmission spectra of the structured membrane are in good quantitative and qualitative agreement with the theory. The developed technique can be used to create an infrared filter for circular polarization.

## Data Availability

Supporting data can be provided upon request by email.
